# Comparison of Serum Urea, Salivary Urea, and Creatinine Levels in Pre-Dialysis and Post-Dialysis Patients: A Case-Control Study

**DOI:** 10.7759/cureus.36685

**Published:** 2023-03-26

**Authors:** Vaishnavi Nagarajan Bhuvaneswari, Hariharan Alexander, Mamatha T Shenoy, Sriramulu D, Suganthy Kanakasekaran, Mohanty Pradipta Kumar, Viveka Murugiah

**Affiliations:** 1 Medicine, Velammal Medical College Hospital and Research Institution, Madurai, IND; 2 Biochemistry, Velammal Medical College Hospital and Research Institution, Madurai, IND; 3 Nephrology, Velammal Medical College Hospital and Research Institution, Madurai, IND

**Keywords:** serum creatinine, serum urea, salivary creatinine, salivary urea, hemodialysis, ckd

## Abstract

Background

Frequent venepuncture for monitoring of serum urea and creatinine in chronic kidney disease (CKD) patients on dialysis will result in venous damage and infection. In this research, we assessed the feasibility of utilizing salivary samples as a substitute for serum samples in determining the levels of urea and creatinine in patients with CKD undergoing dialysis.

Methods

The study participants included 50 patients diagnosed with CKD undergoing hemodialysis and an equal number of apparently healthy individuals. We measured the serum and salivary levels of urea and creatinine in normal subjects. CKD patients were also subjected to similar investigations both before and after hemodialysis.

Results

In our study, we found that the mean value of salivary urea and creatinine are significantly elevated in the case group (salivary urea: 99.56 ± 43.28 mg/dL, salivary creatinine: 1.10 ± 0.83 mg/dL) as compared to the control group (salivary urea: 33.62 ± 23.84 mg/dL, salivary creatinine: 0.15±0.12 mg/dL, p value: <0.001). There was a statistically significant reduction in the mean value of salivary urea and creatinine in the post-dialysis sample (salivary urea: 45.06 ± 30.37 mg/dL, salivary creatinine: 0.43±0.44 mg/dL) compared to the pre-dialysis sample (salivary urea: 99.56 ± 43.28 mg/dL, salivary creatinine: 1.10 ± 0.83 mg/dL; p value: <0.001) in the case group. The salivary urea is significantly positively correlated with serum urea (r value: 0.366, p value: 0.009). But there is no significant correlation seen between salivary and serum creatinine. We have created a cut-off for salivary urea (52.5 mg/dL) to diagnose CKD which has a good sensitivity (84%) and specificity (78%).

Conclusion

The results of our study suggest that the estimation of salivary urea and creatinine could serve as a non-invasive, alternative marker for the diagnosis of CKD, and benefit in risk-free monitoring of their progress before and after hemodialysis.

## Introduction

Chronic kidney disease (CKD) is defined as abnormalities of the kidney structure or function, present for >3 months with health implications [1]. It is prevalent in India, with estimates ranging from 13% to 15.04% [2]. Unfortunately, the incidence of this condition is on the rise and continues to increase annually. Patients with CKD develop several complications like hypertension, electrolyte abnormality, anaemia, alteration in serum calcium and phosphorus concentration due to non-conversion of 25 hydroxycholecalciferol to 1, 25 dihydroxycholecalciferol, acid-base abnormality, reduced excretion of waste products like urea and creatinine which result in multiorgan dysfunction [3-4].

Patients suffering from CKD stage 5 need prolonged renal replacement therapy but the ultimate treatment is a renal transplant. The reduced availability of matching donor organs requires the patient to wait for a long period of time. During this period, the patient undergoes dialysis to prevent complications due to CKD [5]. In dialysis patients, serum urea and creatinine are measured both before and after dialysis to assess the function of the kidney. Since patients with CKD need to undergo frequent dialysis, repeated venous sample collection for measurement of serum urea and creatinine has to be done which is an invasive technique and can cause damage to the blood vessel and infection in the puncture site [6]. So an alternate non-invasive method to assess renal function is required.

Research has demonstrated that it is possible to determine the levels of urea and creatinine in salivary samples [7]. Sample collection is a non-invasive and economic procedure. In patients with hemophilia and inaccessible peripheral veins, salivary samples can be used as an alternate method of sample collection to assess the renal function [6]. There is a paucity of data for the replacement of serum estimation of urea and creatinine with salivary urea and creatinine measurement. We planned this study to validate the utility of salivary samples in place of serum to measure urea and creatinine, before and after dialysis.

## Materials and methods

This is a case-control study that was conducted by the Department of Biochemistry in collaboration with the Nephrology department at Velammal Medical College Hospital and Research Institute, Madurai, Tamil Nadu (a tertiary care hospital). The study was approved by the Institutional Ethics Committee (IEC) of Velammal Medical College Hospital and Research Institute (VMCIEC/36/2022). The study was conducted for a duration of three months - from July 2022 to September 2022. The sample size was calculated based on the study by Jalagam et al. in which the correlation between serum creatinine and salivary creatinine was 0.449 [3]. The minimum sample required to conduct the study was ‘n’. n = [(Z1-q2+Z1-p)2/[1/2ln(1+r/1-r)]2]+3 at 95% confidence interval and 80% precision (n=37). The minimal number of samples required for conducting the study was 37 in each group. In our study, we kept the sample size as 50 CKD patients and 50 healthy subjects. Subjects who came for a master health check-up at our college without a history of diabetes mellitus, hypertension, and renal disease were included in the control group and patients with CKD who presented to the Nephrology department for hemodialysis were included in the case group. Patients with mouth ulcers are excluded from both the groups. Written informed consent was obtained from all subjects.

In our hospital, the age range of patients seeking hemodialysis treatment was between 35 and 60 years. Therefore, we kept the age range between 35 to 60 years in both the case and control groups. In the case group, 3 ml of whole blood was collected in a clot activator container before and after hemodialysis; 3 ml of whole blood was collected once from the control group. Also, 5ml of spot mid-stream urine was collected in a sterile container from the control group for estimation of the albumin creatinine ratio to rule out early renal impairment.

For both the case and control groups, the salivary sample collection method used was the spitting method, as described in the study by Temilola et al. [8]. To minimize the impact of diurnal variation, saliva collection was conducted between 9:00 AM and 12:30 PM. Participants were given instructions to abstain from food and drink for at least 90 minutes prior to collection and to rinse their mouths with water to clear out any residual saliva. Samples were collected using the spitting method and involved the participant filling a 2 mL sterile container with whole saliva. They were asked to sit comfortably with their eyes open and heads slightly tilted forward, avoiding any oral movements or swallowing during the collection process. The participant collected saliva by spitting the pool of saliva into the container every 60 seconds or just before feeling the urge to swallow, repeating this process until 2 mL of whole saliva was obtained. The saliva samples were subjected to centrifugation at 1000 rpm for 10 minutes to obtain the supernatant. The supernatant obtained after centrifugation was immediately used to measure the levels of urea and creatinine in saliva.

Urea levels in both serum and saliva were determined using the urease method, while creatinine levels in both serum and saliva were assessed through the enzymatic method. Urinary creatinine for calculating urinary albumin creatinine ratio was estimated by the enzymatic method. Urinary microalbumin was estimated by bromocresol green method. The urinary albumin creatinine ratio was calculated by using estimated urinary microalbumin. All analysis was done using TBA-120 FR (Toshiba Medical Systems, Tokyo, Japan) fully automated chemistry analyzer after following all quality control protocols according to the National Accreditation Board for Testing and Calibration Laboratories (NABL) norms.

Statistical analysis

All parametric data were expressed as mean ± standard deviation and non parametric data were expressed as median and interquartile range. Independent student t-test was used to find out the difference in the mean values of serum urea, serum creatinine, salivary urea, and salivary creatinine in the case and control groups. Paired student t-test was done to find out if there was a difference in mean value between the pre-dialysis and post-dialysis values of serum and salivary urea and creatinine. Pearson correlation was done between serum and salivary urea, serum and salivary creatinine. To create a cut-off for salivary urea and salivary creatinine, ROC was done. Sensitivity, specificity, positive predictive value (PPV), negative predictive value (NPV), positive likelihood ratio, and negative likelihood ratio were calculated. All analysis was done using SPSS, version 16 (IBM Corp., Armonk, NY).

## Results

The study population consists of two groups; 50 healthy subjects in the control group and 50 CKD patients in the case group. In the case group, 68% had hypertension and 22% had diabetes mellitus. There was no difference in the mean age between the case and control groups. The case group were predominantly (88%) males, whereas in the control group, 52% were male and 48% female. The BMI was significantly higher in the control group compared to the case group (p value: 0.009). The average frequency of hemodialysis in patients with CKD was two times per week (Table 1).

**Table 1:** Comparison of demographic and baseline characteristics between the case and control groups *p value less than 0.05 was considered statistically significant

Variables		Case (pre-dialysis) (n=50)	Control (n=50)	p value
Age (in years)	Mean ±SD	46.78 ± 7.82	48.90 ± 8.29	0.191
Male: Female		44:6	26:24	-
History of Diabetes Mellitus		22% (11/50)	-	-
History of hypertension		68% (34/50)	-	-
Systolic Blood Pressure (mmHg)	Median (25^th^ Percentile,75^th^ Percentile)	140 (100,200)	118 (110,128)	< 0.001^*^
Diastolic Blood Pressure (mmHg) (Median, interquartile Range)		90 (70,100)	80 (72,86)	< 0.001*
Body Mass Index	Mean ±SD	22.80 ± 4.40	25.36 ± 5.19	0.009^*^
Frequency of haemodialysis		Twice / week	-	-
Urinary Albumin creatinine ratio (mg/g)	Mean ± SD	-	17 ± 7.75	-
Serum Urea (mg/dL)		108.60 ± 26.70	23.16 ± 7.62	< 0.001^*^
Serum Creatinine (mg/dL)		10.07 ± 3.82	0.74 ± 0.23	< 0.001^*^
Salivary Urea (mg/dL)		99.56 ± 43.28	33.62 ± 23.84	< 0.001^*^
Salivary Creatinine (mg/dL)		1.10 ± 0.83	0.15 ± 0.12	< 0.001^*^

**Figure 1:**
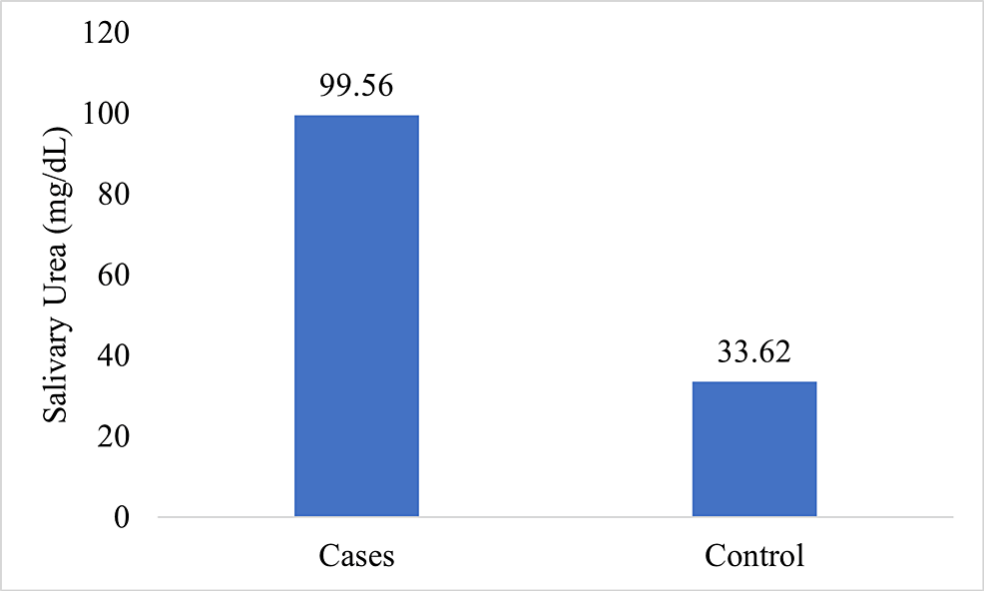
Comparision of salivary urea in the case group (pre-dialysis) and control group

**Figure 2:**
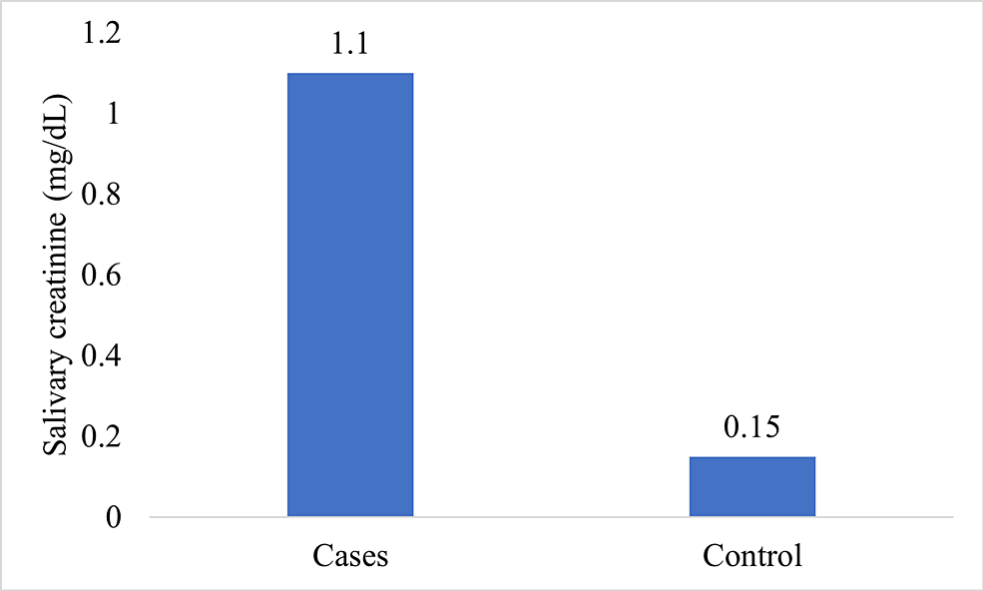
Comparision of salivary creatinine in the case group (pre-dialysis) and control group

**Figure 3:**
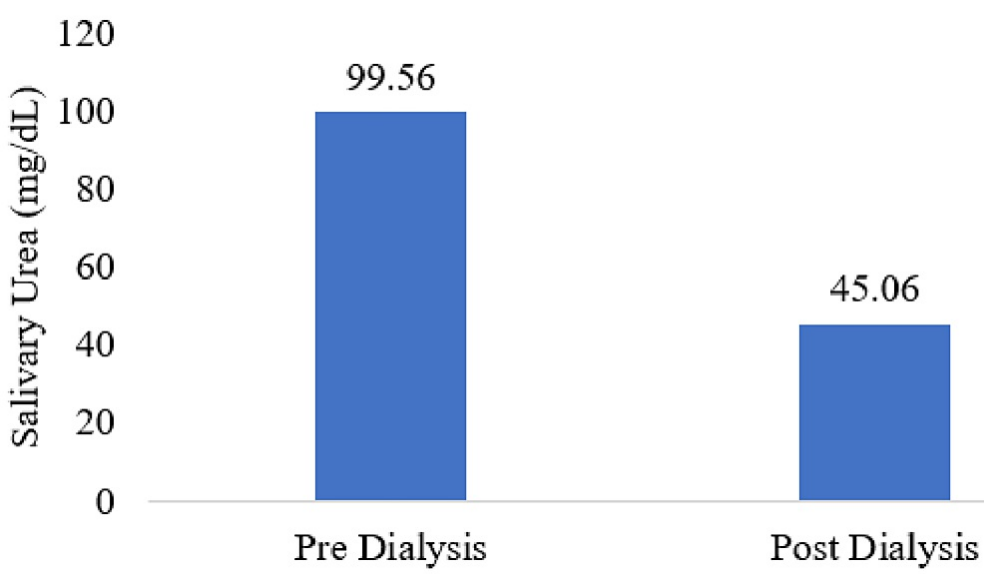
Comparison of salivary urea in pre and post-dialysis chronic kidney disease patients

**Figure 4:**
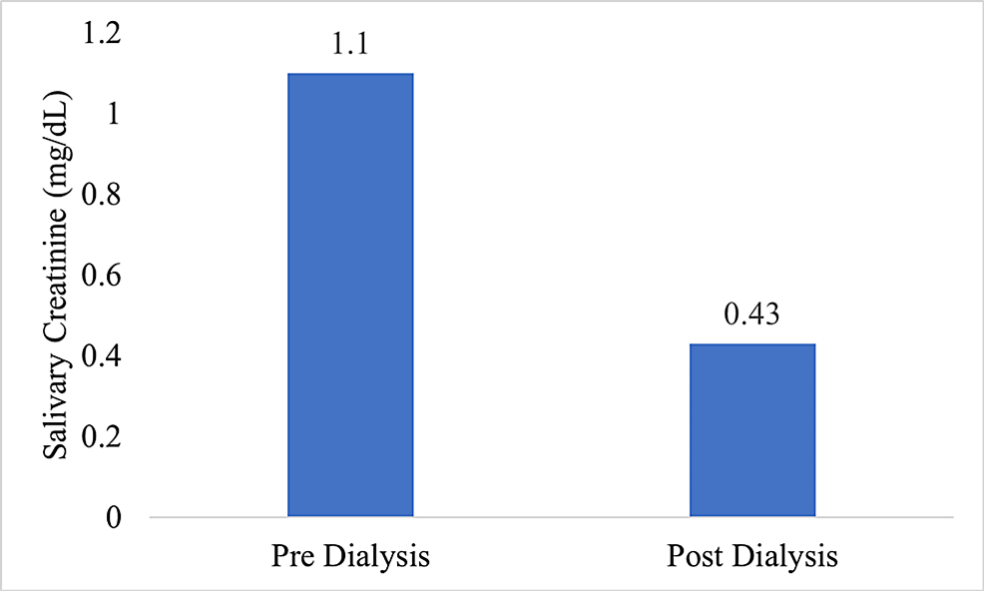
Comparison of salivary creatinine in pre and post-dialysis chronic kidney disease patients

Salivary urea and salivary creatinine displayed significant statistical difference in the case (pre-dialysis) and control group (Figures 1-2). In CKD, the pre-dialysis salivary and serum urea as well as creatinine concentration were higher than post-dialysis salivary and serum urea and creatinine concentration and it was statistically significant (Table 2, Figures 3-4).

**Table 2:** Comparison of salivary and serum urea and creatinine in pre and post-dialysis chronic kidney disease patients *p value less than 0.05 was considered statistically significant

	Pre-dialysis	Post-dialysis	p value
Serum Urea	108.60 ± 26.70	48.54 ± 17.48	< 0.001^*^
Serum Creatinine	10.07 ± 3.82	4.91 ± 1.98	< 0.001^*^
Salivary Urea	99.56 ± 43.28	45.06 ± 30.37	< 0.001^*^
Salivary Creatinine	1.10 ± 0.83	0.43 ± 0.44	< 0.001^*^

**Figure 5:**
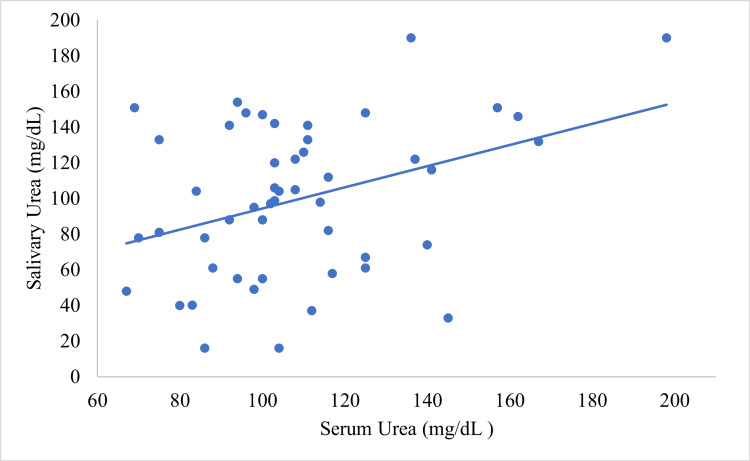
Scatter plot showing correlation between serum and salivary urea in pre-dialysis

Figure 5 shows the correlation between serum urea and salivary urea in CKD patients in the pre-dialysis sample and there was a statistically significant positive correlation (r value: 0.366; p value; 0.009). There was no significant correlation seen between serum creatinine and salivary creatinine in the pre-dialysis sample (r value:0.232;p value:0.105).

**Figure 6:**
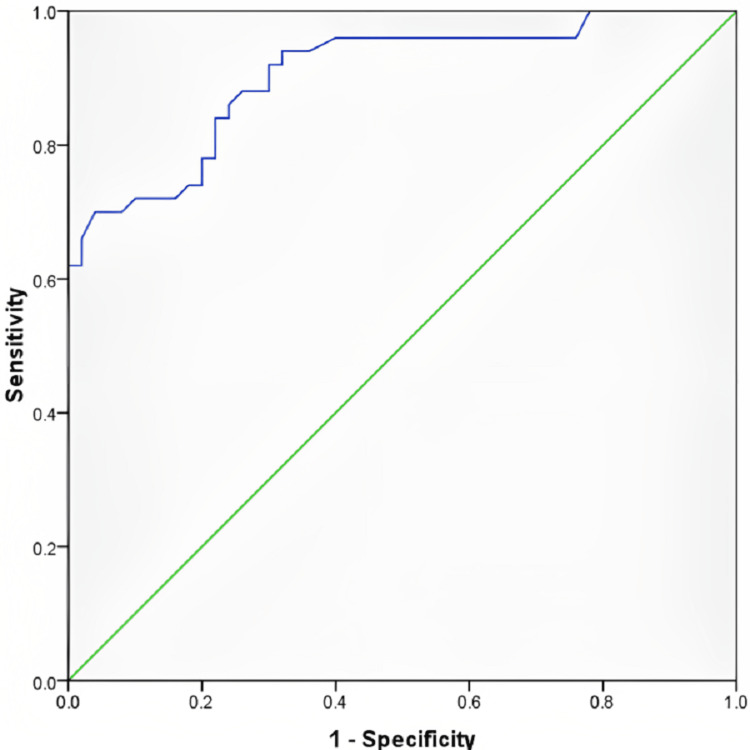
ROC curve using salivary urea for detecting chronic kidney disease ROC: Receiver Operative Characteristic

**Figure 7:**
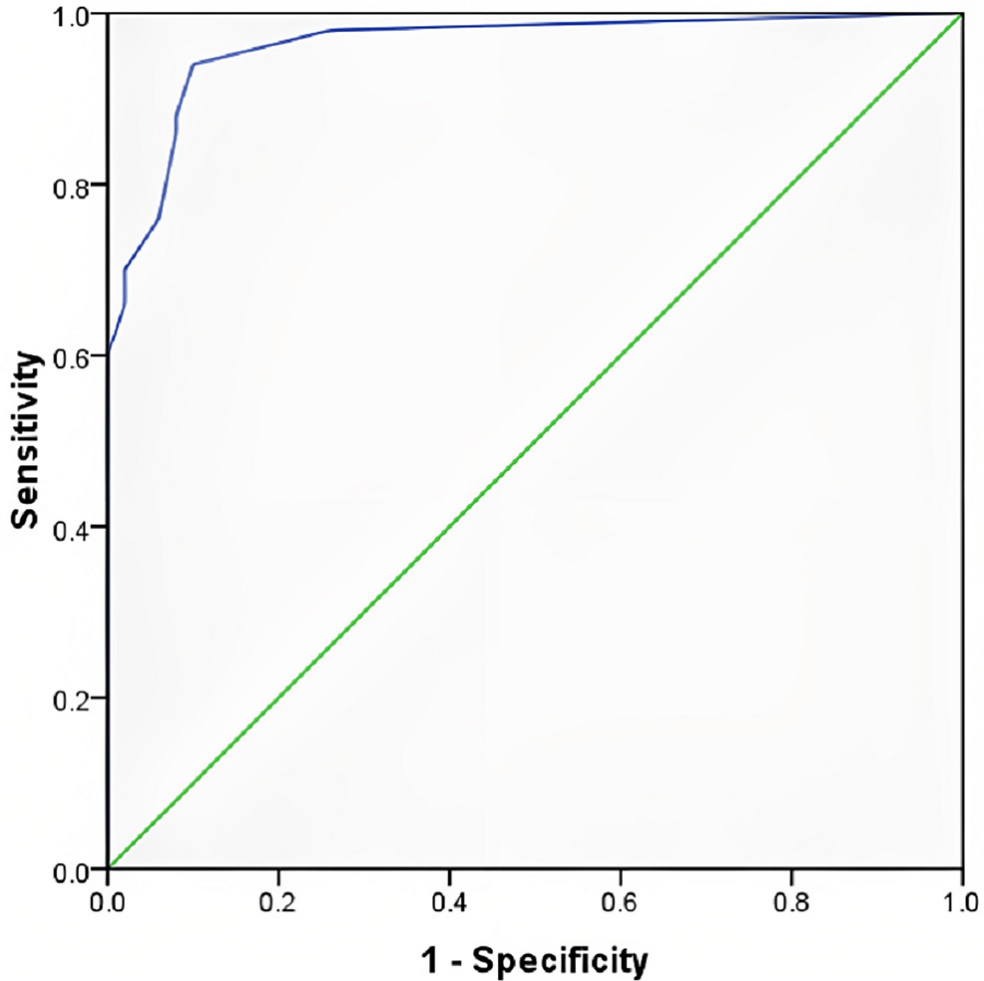
ROC curve using salivary creatinine for detecting chronic kidney disease ROC: Receiver Operative Characteristic

The receiver operating characteristic (ROC) curve was used for discriminating between cases and controls groups by salivary urea and salivary creatinine of pre-dialysis samples (Figures 6-7). The area under the ROC curve (AUROC) for salivary urea was 0.905 (p value: < 0.001) and for salivary creatinine, AUROC of 0.962 (p value: < 0.001) was obtained. Salivary urea showed an optimum cut-off value of 52.5 with sensitivity of 84% and specificity of 78%; salivary creatinine showed an optimum cut-off value of 0.25 with sensitivity of 94% and specificity of 90%. The PPV and NPVs, and positive and negative likelihood ratios of salivary urea and salivary creatinine are shown in Table 3.

**Table 3:** Performance characteristics of salivary urea and salivary creatinine in diagnosing chronic kidney disease *p value less than 0.05 was considered statistically significant AUC: area under the receiver operating characteristic (ROC) curve; PPV: positive predictive value; NPV: negative predictive value.

	Salivary Urea	Salivary Creatinine
AUC	0.905	0.962
p value	< 0.001^*^	< 0.001^*^
Cut-off (mg/dL)	52.5	0.25
Sensitivity (95 % CI)	84 % (70.89 – 92.83)	94 % (83.45-98.75)
Specificity (95 % CI)	78 % (64.04 – 88.47)	90 % (78.19-96.67)
PPV (95% CI)	79.25 % (69.08-86.71)	90.38 % (80.32-95.59)
NPV (95% CI)	82.98 % (71.75-90.34)	93.75 % (83.30-97.83)
Positive Likelihood Ratio	3.82 (2.23-6.52)	9.40 (4.08-21.65)
Negative Likelihood Ratio	0.21 (0.11 – 0.39)	0.07 (0.02-0.20)

## Discussion

Frequent monitoring of serum urea and serum creatinine is required for CKD patients on dialysis and repeated puncturing of veins for sample collection for the estimation of serum urea and serum creatinine can result in damage to veins [6]. So, there is a need for a non-invasive method to estimate urea and creatinine levels in CKD patients.

This study was intended to analyze an alternate non-invasive method to estimate urea and creatinine in CKD patients undergoing hemodialysis. In our study, there was no significant difference in the mean age between the case and control groups. Hence, age does not influence the outcome of the study. The percentage of males was high in the case group in our study. This finding is contradictory to the result from another study which stated that the prevalence of CKD was high in the female gender [9]. Our study population had higher males in the case group. The lesser number of females could be due to cultural and economic factors among the general population which has led to females having lesser access to dialysis [10]. The percentage of hypertension and diabetes mellitus in the case group was 68% and 22%, respectively. Several studies have stated that hypertension and diabetes mellitus are two major causes of CKD and the same has been reflected in our study [11-12]. Our study shows that BMI was higher in the control group compared to the case group. This could be due to muscle wasting seen in patients with CKD [13].

Our study upholds the findings noted previously by other studies that salivary urea and salivary creatinine levels are significantly high in CKD patients when compared to the control group [14]. Salivary urea and salivary creatinine concentration were significantly higher in the pre-dialysis sample compared to the post-dialysis sample and a positive correlation is seen between serum urea and salivary urea in CKD patients. A similar finding was observed in a previous study [15]. In our study, there was no correlation seen between serum and salivary creatinine levels. This might be due to the low concentration of creatinine seen in salivary samples. But even though the salivary creatinine is low, there is a statistically significant difference seen between cases and controls. There is a statistically significant difference seen in salivary creatinine between pre-dialysis and post-dialysis patients. Increased serum urea and creatinine concentrations in CKD patients may result in the diffusion of these analytes from serum into saliva, indicating that saliva could be an alternative route of excretion in cases of renal impairment. Elevated concentrations of urea and creatinine in saliva due to CKD can cause a variety of complications, including dry mouth, uremic breath, and the formation of tongue coating, among others [16]. Since following dialysis, blood levels of urea and creatinine were reduced, and salivary concentration of urea and creatinine was also reduced [6]. ROC was done to create a cut-off for salivary urea and creatinine and we observed a good sensitivity and specificity for the created cut-off.

Limitations

The study had a few limitations which could be considered in future studies. Since the results obtained from this study was from a single center, further multi-centric studies are warranted to know whether the findings apply to the general population. Moreover, the present study does not account for widespread changes in practice between different health centers. In this regard, future multi-centric studies could provide much-needed external validity to support the variance in population and clinical practice. Lack of commercially available kits which use saliva as a sample for estimating urea and creatinine provides scope for future studies. Furthermore, recruitment and matching for gender among control subjects with cases would reduce the variance in the parameters of interest and improves statistical efficiency. Other studies in this line could also consider employing a highly sensitive creatinine assay to explore the correlation between serum and salivary creatinine levels. Statistically, the sample size was estimated and found to be 37 subjects in each group, namely CKD and healthy subjects. We enrolled 50 CKD patients as cases and 50 apparently healthy adults as controls, thus our findings are statistically validated. The study, if conducted in a larger population, can help confirm our findings.

## Conclusions

Patients undergoing dialysis face an increased risk of venous injury and infection due to frequent venepuncture for urea and creatinine monitoring. This study aims to explore the feasibility of utilizing salivary samples as a safer and non-invasive substitute for serum samples in estimating urea and creatinine levels in CKD patients undergoing dialysis. Of importance, very much similar to the serum urea and creatinine, a clear distinction was observed between the levels of salivary urea and creatinine between CKD patients and healthy subjects. Notably, we identified that salivary urea and creatinine declined in CKD subjects after they underwent hemodialysis. In particular, salivary urea levels markedly correlated with serum urea levels and exhibited excellent sensitivity and specificity for detecting CKD. Hence, salivary urea could serve as a non-invasive marker for diagnosing CKD in patients undergoing dialysis. Further multi-centric studies with a larger group of subjects could validate the application of salivary urea and creatinine estimation as a safer method for monitoring the recovery of renal function in patients undergoing dialysis.
